# Investing in Communities: Evaluating the Added Value of Community Mobilization on HIV Prevention Outcomes Among FSWs in India

**DOI:** 10.1007/s10461-013-0626-6

**Published:** 2013-10-16

**Authors:** Anne Sebert Kuhlmann, Christine Galavotti, Philip Hastings, Pradeep Narayanan, Niranjan Saggurti

**Affiliations:** 1 Washington University in St. Louis, One Brookings Drive, St. Louis, MO 63130 USA; 2 CARE USA, 151 Ellis Street, Atlanta, GA 30303 USA; 3 Far Harbor LLC, Austin, TX USA; 4 Praxis Institute for Participatory Practices, New Delhi, India; 5 Population Council, New Delhi, India

**Keywords:** Community participation, Community mobilization, HIV prevention, Female sex workers, Evaluation methodology

## Abstract

Community mobilization often requires greater time and resource investments than typical interventions, yet few evaluations exist to justify these investments. We evaluated the added benefit of community mobilization on HIV prevention outcomes among female sex workers (FSWs) using a composite measure of volunteer participation in program committees by FSWs. After adjusting for treatment propensity, we used multilevel structural equation modeling (MSEM) to test our program theory. We hypothesized that stronger community mobilization would be associated with increased levels of consistent condom use and with increased levels of perceived fairness, mediated by psychosocial processes. Community mobilization had an indirect effect on consistent condom use mediated through social cohesion and an indirect effect on perceived fairness mediated by collective efficacy. Our results suggest higher levels of community mobilization help improve condom use and reduce perceived discrimination beyond the effects of the core HIV intervention program. We recommend further testing of this model.

## Resumen

Movilización de comunidad requiere más tiempo e inversión de recursos que una típica intervención, pero muy pocas evaluaciones existen para justificar estas inversiones. Se evaluó el beneficio de movilización de comunidad en la prevención de VIH en sexoservidoras usando un indicador compuesto de participación de voluntarios en comités de programa por sexoservidoras. Después de ajustar por propensión de tratamiento, se usó una ecuación de múltiple niveles estructural para probar nuestra teoría de programa. Nuestra hipótesis es que una movilización de comunidad más fuerte estuviese asociada con un nivel aumentado de uso de condones consistente con niveles aumentados de imparcialidad percibida, mediada por procesos psicosociales. Movilización de comunidad tuvo un efecto indirecto en el uso de condones consistente mediado por cohesión social y un efecto indirecto en la percibida imparcial mediado por eficacia colectiva. Nuestros resultados sugieren que niveles elevados de movilización de comunidad ayudan a mejorar uso de condones y reducir discriminación percibida más allá de los efectos de la intervención del programa de VIH. Recomendamos pruebas adicionales de este modelo.

## Introduction

Although most HIV prevention program implementers recognize that community participation and mobilization is important to program success, community mobilization as an intervention strategy usually necessitates greater and longer term investments of financial and human resources than more traditional HIV prevention interventions. While more traditional, targeted HIV prevention interventions such as peer education and outreach with condom distribution have shown positive impacts on HIV prevention behaviors among individuals of high-risk groups [1–3], the impacts of community mobilization have been less well documented. Community mobilization seeks to engage participants in a way that increasingly allows them to make decisions and shape their own lives [4]. Spurring and supporting this deepening engagement often requires significant human resources and time investments to facilitate development of decision-making, leadership, and management skills in marginalized populations. In an era of increasingly limited resources, funders and policy makers are particularly keen to see evidence that community mobilization produces substantially better HIV prevention outcomes or additional benefits to justify these larger investments. And, though there is some evidence that community mobilization improves HIV prevention outcomes [5–7], how community mobilization works to produce these outcomes remains virtually unexplored.

Understanding how community mobilization may contribute to program success is complicated by several factors. First, there is no common definition of community mobilization—interventions range from community education and sensitization to community-led structural interventions—and effects of such a wide range of interventions are likely to vary just as widely. Further, few authors have laid out a conceptual model or program theory that explains how community participation and mobilization is expected to lead to specific outcomes: what is the mechanism of effect? Thus, we lack consensus on what intervention activities constitute a community mobilization intervention, what a “mobilized community” looks like, and how that mobilization is expected to result in the desired program outcomes. Evaluation is further hampered by the fact that community mobilization is an inherently complex and dynamic process that occurs over time, evolving in ways that may be heavily dependent on the community and the context in which it is undertaken.

Nevertheless, community mobilization continues to be an important component of many public health interventions, and has been shown to have an effect on a variety of sexual, reproductive, maternal, and child health outcomes. Researchers have found evidence of a significant, positive impact of community mobilization on reducing child stunting in Bangladesh [8], reducing neonatal mortality rates in Nepal [9], India [10], and Malawi [11], and some evidence for increasing birth planning and emergency transportation in Bangladesh [12]. Thus, knowledge gained about how community mobilization works may be applicable to a variety of sexual, reproductive, maternal, and child health areas.

Avahan, the India AIDS Initiative funded by the Bill and Melinda Gates Foundation, works with a number of high-risk groups in the six Indian states with the highest HIV prevalence. Avahan funds one or two state lead partners (SLPs) in each state who then fund and work with hundreds of local non-governmental organizations (NGOs) to implement the intervention [13]. Avahan’s scale of reaching nearly 200,000 female sex workers (FSWs) in 83 districts across 6 states with a combined population of 300 million [13] along with its work with a large range of high-risk groups (e.g., FSWs, male clients of sex workers, truck drivers) has been unparalleled in community mobilization and HIV prevention. Thus, Avahan provides a unique opportunity to evaluate the potential added value of a community mobilization intervention on HIV prevention outcomes as well as to examine the impact of community mobilization on additional outcomes that would not be expected from more traditional, targeted intervention strategies.

Avahan planners carefully laid out a multi-level population impact assessment early on in the program [14]. For a variety of reasons, however, evaluating the community mobilization component was not included as part of this initial impact assessment plan. Early results from the monitoring data and staff perceptions nevertheless suggested that the community mobilization intervention activities played a critical role in Avahan: community members (i.e., members of the high-risk group targeted by the intervention, in this case FSWs) helped to map high-risk populations in the districts, advised on key program decisions like the location of health clinics and drop-in centers, and worked as peer educators contacting over 70 % of the high-risk population in intervention districts on a monthly basis [13, 15–17].

The challenge, therefore, was how to design and implement an evaluation of Avahan’s community mobilization efforts after initial program implementation had already begun. We chose to use theory-based evaluation [18]. We articulated a program theory to describe how Avahan’s intervention spurs and supports the community mobilization process and how that process leads to enhanced HIV prevention outcomes and additional benefits, and then developed a phased evaluation plan to test this program theory (see Galavotti et al. [4] for further information on the program theory). In this paper, we present results from the first phase of the evaluation assessing the added value of community mobilization on HIV prevention outcomes among FSWs in Andhra Pradesh, India.

## Methods

### The Model and Hypotheses

The primary goal of Avahan is to reduce HIV infection by promoting risk reduction behaviors and supporting an enabling environment among high risk groups. Across Avahan, a common core of targeted intervention activities are implemented including drop-in centers, peer outreach, condom distribution, crisis response and services for sexually transmitted infections (STIs). The community mobilization component of Avahan consists of the work to engage sex workers in program activities, from encouraging participation in initial community mapping and outreach and membership in project committees, to facilitating their management of crisis response teams and their taking leadership roles in formal and informal community based organizations; the greater the participation, engagement and ownership of the intervention by the FSWs, the more “mobilized” the community. The goal of this community participation is to mobilize the community of sex workers so that they can increasingly make decisions, influence their environment, and shape their lives in ways that support their health and well-being, including prevention of HIV infection.

Our program theory describes how this participation sets in motion a number of causal processes (mediators) through which the intervention ultimately leads to better program outcomes. These mediating variables reflect the expanding interpersonal, social and political space that sex workers inhabit as mobilization unfolds. The process begins when sex workers claim their identity as sex workers and begin to see themselves as part of a community [19]. This *identification* is characterized by increased self-confidence, individual agency (e.g., I can refuse a client when tired), and self-efficacy. As social cohesion and connectedness grow, sex workers begin to understand that they are part of a group that faces common concerns, experiences and needs [20]. This *collectivization* is characterized by a strong sense of collective identity, collective efficacy, agency and action (e.g., FSWs can work together to achieve goals, stand up for each other), and social cohesion (e.g., trust, reciprocity, belongingness). (Please see Galavotti et al. [4] for a more detailed discussion of the program theory and how the Avahan intervention spurs and supports the community mobilization process.)

**Fig. 1:**
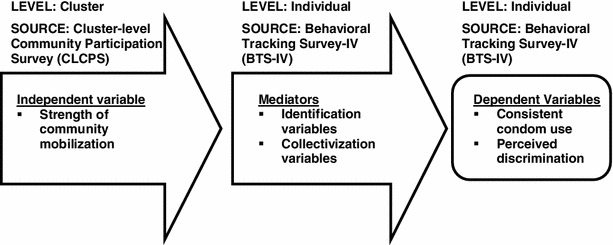
Model for evaluation of community mobilization: level and source of data for key constructs

Based on our program theory, we constructed a model (see Fig. 1) to test the hypothesis that higher levels of community mobilization would result in increased identification and collectivization which in turn would lead to more positive HIV prevention outcomes. This model does not represent the full program theory as we could only test those variables in the model for which we could collect valid data.

### Evaluation Design

Our evaluation focused on FSWs in the state of Andhra Pradesh within a district served by one of Avahan’s implementing partners.^1^ Conducting the evaluation in areas where a single state lead implementing partner (SLP) was delivering the intervention helped minimize intervention variation due to implementation style of the SLP; it also minimized contextual effects that might arise from geographic and cultural differences across states in India.

To achieve adequate sample size, we sampled 104 geographic clusters within the district and then measured strength of the treatment (i.e. level of community mobilization) at the cluster level. These clusters were small geographic units under the responsibility of one outreach worker (ORW) who served ~250 FSWs. This unit then became the area in which we measured “strength of community mobilization”, as well as our primary sampling unit for the survey of sex workers. Although all 104 clusters implemented the same core program activities, we anticipated that the level of community mobilization, i.e. volunteer participation and engagement of FSWs in those activities, would vary.

Next, we defined our measure of treatment strength: level of community mobilization in the cluster. Previous researchers have shown positive relationships between self-reported exposure to the program (as a proxy for community mobilization) and both psychosocial and behavioral outcomes [21, 22]. Most recently, a study of Avahan in Karnataka showed a positive effect of self-reported exposure to the community mobilization intervention on condom use and uptake of HIV/STI services outcomes [23]. However, self-reported measures of treatment exposure are subject to recall bias, as well as selection bias, since those who participate may be different from those who do not. Further, since community mobilization is a community-level, not an individual-level, intervention, it makes sense to measure treatment strength at the community rather than the individual level.

To create an unbiased measure of treatment strength at the community level, we needed an independent measure of the level of community mobilization in the cluster. Although extensive program monitoring data were available to confirm implementation of intervention activities, community mobilization is not the activities themselves but rather the level of participation, engagement and ownership of the community in those activities. Identifying reliable measures of “mobilization” that were collected through routine program monitoring data proved impossible.

Therefore, we collected data from the ORWs responsible for the defined clusters and validated these data by reviewing program records (described more in “Data Sources and Collection” section). Our measure of the strength of the intervention assessed the level of participation in specific program committees by volunteer FSWs (vs. paid staff or paid peers) in a given geographic cluster. Level of participation is an indicator of interest and involvement, and *volunteer* participation suggests an even greater level of commitment and ownership. We were thus able to characterize clusters in terms of the strength of the treatment: community mobilization.

Still, we faced further challenges in the design of the evaluation. Avahan was designed to be implemented at scale (saturating districts with the intervention) and no control sites were planned. Therefore, our only option was to investigate whether the intervention strength would exhibit a dose–response relationship to our outcomes of interest among the “treated” clusters. A vulnerability of this approach is that selection bias can arise among clusters, since individuals were not randomly assigned to levels of the intervention—treatment strength in a cluster could only be observed, not controlled.

The classic strategy for minimizing selection bias is to employ a randomized controlled design, which is not typically available for field interventions. To address this issue, we used a propensity score method to reweight the original sample such that the pre-intervention confounding variables were unrelated to level of treatment in the reweighted sample. Propensity score methods can be effective in minimizing selection bias by creating a weighted pseudo-sample (conditioned on the observed confounders) which somewhat approximates a randomized design. The details of this adjustment are further described in the “Analysis Process” section.

We could not use data from previous surveys in the district as a baseline for our behavioral outcomes and mediators since those data were collected using a different sampling frame. Our outcome measures—consistent condom use with clients and perceived discrimination—and our mediating variables were therefore collected via an independent survey of FSWs in the same 104 geographic clusters.

This cross-sectional, dose–response design, using an independent measure of strength of treatment at the cluster level, allowed us to explore whether (high) community mobilization leads to improved program outcomes above and beyond what would be expected from the core Avahan HIV interventions (no/low mobilization). Using multi-level structural equation modeling (MSEM) coupled with propensity score reweighting to adjust for selection bias, our approach attempted to simulate a randomized dose–response trial to estimate non-biased treatment effects.

### Data Sources and Collection

#### Cluster-Level Community Participation Survey (CLCPS)

Because self-reported measures of exposure to, and participation in, an intervention program suffer from problems of recall and social desirability biases that tend to conflate self-reported program exposure with positive outcomes (especially when such data are collected within the same survey), we measured strength of community mobilization through the CLCPS, which provided a profile of community participation within the clusters. ORWs in 104 clusters were interviewed using a series of questions to measure community participation in program implementation, program management, crisis response, decision-making, and program activities. Data ranged from information about proportion of community members planning, implementing and overseeing program activities, to governance processes, leadership and ownership. These variables served as the basis for a composite intervention strength variable characterized by the level of community participation in each cluster. We validated the information provided by the ORWs through structured interviews with peer educators and ORWs responsible for the cluster and detailed reviews of organizational documents such as meeting minutes, micro-planning documents detailing monthly outreach contacts, organizational by-laws, and annual reports. The data collection tool and process for the CLCPS were based on a detailed participatory monitoring tool being used in a select number of Avahan districts [24].

#### Behavioral Tracking Survey (BTS-IV)

In the same sample of 104 clusters where the CLCPS was conducted, individual FSWs were randomly sampled for participation in a BTS-IV using a two-stage sampling procedure. In the first stage, a fixed number of hotspots within each cluster were selected via the proportion to population size (PPS) procedure. In the second stage, we selected participants using either conventional cluster sampling from non-public places (e.g., brothels) or time-location cluster sampling from public places (e.g., streets, parks, highways). We collected information on the number of FSWs per hotspot and the times when they gathered for sex work in order to weight the sample. More detail on the sampling process for this survey has been published elsewhere [25]. A total of 1,986 FSWs participated in the BTS-IV, of 2,389 sampled, for an unweighted response rate of 83.1 %.

The BTS survey has been used across Avahan and modified several times over the last few years. Previous rounds of the BTS in Andhra Pradesh were conducted using a different sampling frame and thus not useful for purposes of this evaluation; however, we did modify and use the tool for this survey. The BTS measures demographics, socioeconomic situation, sex work history, condom use, perceptions of sex worker solidarity and efficacy, participation in FSW organizations and events, and self-reported exposure to the intervention. For the BTS-IV, we added a number of variables specific to this evaluation, including time since first exposed to the Avahan intervention, self-efficacy scales for condom use and for service utilization, contraceptive use, a social cohesion scale validated for use with sex workers [26], and a validated depression measure [27].

#### Ethical Considerations

The overall study design and questionnaires were reviewed and approved by the institutional review boards of Family Health International and the Karnataka Health Promotion Trust. Oral consent was obtained from all respondents prior to participation in the interview, and steps were taken to ensure their confidentiality. For ethical reasons, only those FSWs who were at least 18 years of age were interviewed. No names or addresses were recorded on the questionnaires. Participants were not provided any compensation for their time in the study but were referred to local project services run by the SLP in the district.

### Measures

#### Treatment Variable

A high rate of community (FSW) volunteers serving on intervention planning, implementation and oversight committees is a critical indicator of community mobilization. Thus, strength of community mobilization was calculated as the average percentage of volunteer FSWs participating on seven program-related committees within the cluster, as compared to the total committee membership (i.e. the ratio of volunteers to volunteers plus paid staff).

#### Dependent Variables

We measured HIV prevention as consistency of condom use with both occasional and regular clients by FSWs in the cluster. We also tested a second outcome variable, perceived discrimination in a variety of public places (reverse coded as perceived fairness), as a measure of an enabling environment conducive for HIV prevention.

#### Mediating Variables

Indicators for *identification* included claiming identity as a sex worker, individual agency to refuse clients when tired and to make decisions about one’s own life, self-efficacy for condom use with clients, self-efficacy for service utilization, self-confidence in obtaining condoms and in giving advice, and mental health. Indicators for *collectivization* included collective identity of attending events where one could be identified as a FSW, collective efficacy for FSWs working together to solve problems and for FSWs working together to achieve goals, collective agency for standing up for those in need, collective action of FSWs working together to demand entitlements, and social cohesion among FSWs in the cluster. Table 1 provides greater detail on our variables and their sources.

**Table 1:** Type, description, and source of variables (weighted means and percentages)

Variable	Description	Items (∝+)	*N*	Min	Max	Mean	SE	Source
Treatment variable								
Strength of community mobilization	Average % of unpaid FSWs serving on seven committees in cluster	1 (–)	104	0.17	0.73	0.45	0.01	CLCPS
Identification mediators								
Claim identity	Willingness to self-identify as a FSW	2 (0.62)	1,943	1.0	4.0	2.90	0.04	BTS-IV
Self-confidence 1	Confidence in obtaining condoms	1 (–)	1,943	1.0	4.0	2.47	0.07	BTS-IV
Self-confidence 2	Confidence in giving advice/opinions	2 (0.66)	1,943	1.0	4.0	2.59	0.04	BTS-IV
Self-efficacy 1	Self-efficacy for condom use with clients	3 (0.77)	1,943	1.0	4.0	2.71	0.03	BTS-IV
Self-efficacy 3^a^	Self-efficacy for service utilization	2 (0.83)	1,943	1.0	4.0	2.61	0.05	BTS-IV
Individual agency 1	Turning away clients if tired	1 (–)	1,940	0.0	3.0	0.99	0.06	BTS-IV
Individual agency 2	Autonomy for personal actions	7 (0.87)	1,943	0.0	1.0	0.67	0.02	BTS-IV
Mental health	Mental health (depression reverse coded)	2 (0.88)	1,943	1.0	4.0	2.98	0.07	BTS-IV
Collectivization mediators								
Collective identity	Attended a public event in last 6 months where could be identified as a FSW	1 (–)	1,943	0.0	1.0	0.67	0.03	BTS-IV
Collective efficacy 1	FSWs would work together if problem affected the group	1 (–)	1,943	1.0	4.0	2.56	0.07	BTS-IV
Collective efficacy 2	FSWs work well together for specific goals	4 (0.75)	1,942	0.0	3.0	1.96	0.03	BTS-IV
Collective agency	Negotiated or stood up for FSW in need	4 (0.76)	1,943	0.0	1.0	0.38	0.02	BTS-IV
Collective action	FSWs come together to demand entitlements	7 (0.80)	1,943	0.0	1.0	0.15	0.02	BTS-IV
Social cohesion	Sharing issues, relying on fellow FSWs	12 (0.69)	1,943	1.3	4.0	3.00	0.04	BTS-IV
Outcome variables								
Condom use with clients, continuous	Mean frequency of condom use with regular clients and occasional clients (1 = never to 4 = always)	2 (0.77)	1,943	1.0	4.0	3.51	0.03	BTS-IV
Condom use with clients, categorical	Low		349			20.46 %	1.76	BTS-IV
	Medium		409			18.59 %	1.73	
	High		1,185			60.95 %	2.43	
Perceived discrimination	FSWs perception of discrimination in public places, such as hospitals, bank, and post offices (reverse coded into perceived fairness)	4 (0.80)	1,943	1.0	4.0	2.66	0.04	BTS-IV
Cluster-level confounder variables								
Length of intervention	Months since intervention started in the cluster	1 (–)	104	25.0	111.0	54.50	2.09	CLCPS
FSW density	Estimated density of FSWs per kilometer in cluster	1 (–)	104	10.0	243.0	64.74	4.27	CLCPS

^a^Indicates variable only used in models of perceived discrimination

+Full sample (*n* = 1,986) unweighted Cronbach’s alpha

### Analysis Process

#### Treatment Propensity Adjustment

Due to the potential bias associated with nonrandom selection of FSWs into clusters with varying levels of community mobilization, we employed a propensity score methodology to reweight the data. Studies have shown that propensity methods can remove up to 90 % of the bias resulting from observable confounders [28, 29]. We therefore identified 18 confounders that might influence selection into clusters and the outcomes of interest. Confounding variables were selected a priori following guidelines provided by Yanovitzky et al. [30]. Before data collection, researchers asked subject matter experts familiar with the target population and prior empirical evidence to identify those variables that might influence both an individual’s selection into the program cluster as well as an individual’s outcomes. A final set of demographic characteristics expected to be stable before program intervention and unlikely to be influenced by the intervention were included in the survey during data collection. This set of variables was then used during propensity modeling to estimate an individual’s probability of selection into treatment quintile. The variables included demographics such as age, marital status, education, income, living and work situations, and number of children.

The confounders were used in separate and combined multinomial logistic regressions to predict treatment quintile as the dependent variable (clusters were ordered by strength of community mobilization to form these quintiles). The inverse of an individual’s predicted probability of being in a treatment quintile (given the full set of confounders) was used to reweight the sample. The sample was trimmed to remove 43 observations with very low probabilities (<5 %) so these cases would not have outsize influence on model results [31]. The multinomial logistic model was then retested with the propensity-weighted data. None of the confounders were significantly related to treatment after reweighting, suggesting that the propensity adjustment successfully removed the effect of these variables as significant sources of bias in the analyses (see Table 2).

**Table 2:** Significance of individual-level confounders from multiple regression predicting participation, before and after propensity adjustment, BTS-IV (original sample *n* = 1,986; trimmed sample *n* = 1,943)

		Original Sample—Participation % regressed on confounders		Propensity—Adjusted, Scaled, Trimmed Sample	
Variable	df	Wald *F* value	*p*	Wald *F* value	*p*
Age in years	1	4.76	0.03*	0.54	0.46
Age at first sex	1	5.48	0.02*	0.77	0.38
Years of education	1	4.93	0.03*	0.49	0.48
Years in sex work	1	6.26	0.01*	0.64	0.43
Marital status	3	1.80	0.15	0.33	0.81
Current living situation	3	1.29	0.28	0.27	0.85
Environment for sex work	2	0.29	0.75	0.15	0.86
Frequency of travel for sex work	2	2.48	0.09	0.38	0.68
Number of places conduct sex work in district	2	9.08	0.00**	1.19	0.31
Number of places conduct sex work outside district	2	3.07	0.05	0.36	0.70
Sources of income	2	0.46	0.63	0.11	0.89
Own a cell phone	1	0.04	0.84	0.00	1.00
Type of location for sex work	3	2.49	0.06	0.22	0.88
First sex work experience was coerced	1	1.13	0.29	0.99	0.32
Know someone with HIV	1	0.02	0.89	0.21	0.65
Currently in debt	1	18.36	0.00**	0.58	0.45
Has children	1	2.34	0.13	1.00	0.32
Has school-aged children	1	3.51	0.06	1.47	0.23

* *p* < 0.05, ** *p* < 0.01

#### MSEM

Our model hypothesized that stronger community mobilization would influence psychosocial variables (indicative of identification and collectivization), and that changes in these mediators would be associated with positive HIV-prevention outcomes. The two cluster-level outcomes were: (1) degree of consistent condom use with clients and (2) level of perceived discrimination. Since the strength of community mobilization (measured by the percentage of volunteer FSW participation on committees) was implemented and measured at the cluster level, our analysis necessarily focuses on cluster-level impacts. Stated another way, strength of community mobilization could not have effects at the individual (within-cluster) level since all members of a cluster are assigned the same community mobilization strength value. During the multi-level modeling process, individually measured variables are separated into within- and between-level variance components; the influences of community mobilization strength are evaluated on these between-level components (analogous to mean rates by cluster).

Models also included two cluster-level covariates, the geographic “density” of FSWs in each cluster (per square kilometer) and the time since the cluster intervention began. In this way we tried to ensure that the estimated effect of increased community mobilization was not conflated with the age of the program or the relative density of the target population.

**Fig. 2:**
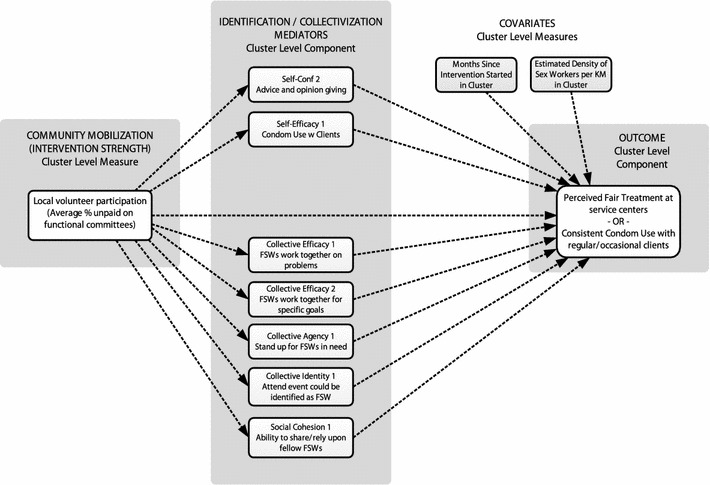
Estimated path model (cluster level). All mediators were allowed to correlate freely in the model; these curved paths are not illustrated for clarity

Given the set of psychosocial scales, modeling proceeded in steps to keep the number of estimated parameters from exceeding the total number of clusters (*n* = 104). First, the identification variables (scales for claiming identity, individual agency, self-efficacy, self-confidence, and mental health) were tested as mediators of community mobilization strength for each outcome. Second, the collectivization variables (scales for collective identity, collective efficacy, collective agency, collective action, and social cohesion) were examined as mediators. Lastly, the significant mediators identified during the first two steps were retained in a combined model in order to estimate a final model for each outcome (see Fig. 2).

This reduced set of identification and collectivization scales was then used to estimate the final models for levels of consistent condom use and perceived discrimination among clusters. Our multilevel path analysis (MSEM with observed rather than latent variables) assumed fixed slopes and random intercepts across the clusters. Mplus version 6.12 [32] was employed to analyze these data, which enabled the simultaneous estimation of parameters for a weighted two-level analysis with cluster-level mediation [33].

## Results

Of the 1,986 FSWs who participated, over half were currently married (57 %) and most had children (81 %). Despite most participants having a source of income in addition to their sex work (78 %), a large majority were in debt (85 %). Over half worked in an urban environment (53 %). With regards to stability of work environment, approximately two-thirds never traveled for work (61 %) and only conducted sex work in the district in which they were interviewed (71 %). A large majority of participants also personally knew someone living with HIV (84 %). Table 3 provides additional information on background characteristics of the participating individuals.

**Table 3:** Demographic characteristics of survey sample, weighted (*n* = 1,986)

Continuous	Mean (SE)
Age in years	29.19 (0.18)
Age at first sex	24.79 (0.18)
Years in sex work	4.11 (0.12)
Years of education (illiterate = −1)	3.40 (0.19)

Categorical	% (SE)
Marital status	
Never married	9.16 (1.15)
Currently married	57.08 (2.16)
Deserted/separated/divorced	19.93 (1.30)
Widowed	13.83 (1.27)
Current living situation	
Living alone	14.06 (1.30)
Living with spouse	43.11 (2.04)
Living with M/F partner/friend/colleague/other FSW	10.50 (1.19)
Living with family	32.33 (1.87)
Environment for sex work	
Urban	53.23 (3.04)
Semi-urban	21.86 (2.85)
Rural	24.91 (3.16)
Frequency of travel for sex work	
Does not travel	60.93 (2.48)
A few times per year	16.17 (1.87)
Monthly or more	22.90 (2.56)
Number of places conduct sex work in district	
Missing/none	62.02 (2.53)
1–2	6.59 (0.82)
3 +	31.39 (2.62)
Number of places conduct sex work outside district	
Missing/none	70.93 (2.27)
1–2	20.69 (1.97)
3+	8.38 (1.22)
Sources of income	
Only sex work	21.86 (2.30)
Sex work plus day laborer	41.61 (2.06)
Sex work plus domestic help/sell veg or flowers/student/bar/salon/other	36.53 (1.83)
Own a cell phone	
No	79.41 (2.18)
Yes	20.59 (2.18)
Type of location for sex work	
Brothel/bar/hotel/dhaba/highway	7.68 (1.38)
Street	44.99 (2.46)
Home	9.95 (1.53)
Phone solicitation/other	37.38 (2.65)
First sex work experience was coerced	
No	87.75 (1.45)
Yes	12.25 (1.45)
Know someone with HIV	
No	16.45 (2.23)
Yes	83.55 (2.23)
Currently in debt	
No	14.71 (1.65)
Yes	85.29 (1.65)
Have children	
No	18.68 (1.41)
Yes	81.32 (1.41)
Have school-aged children	
No	28.40 (2.08)
Yes	71.60 (2.08)

Even though a single SLP led implementation in all clusters, strength of community mobilization varied considerably across clusters. The rate of participation by volunteer FSWs on program committees within the cluster (strength of community mobilization) ranged from 17 to 73 %, with an average of 45 % across the clusters.

Overall, our scales measuring the identification and collectivization mediators had good reliability (see Table 1) and indicate a sense of identification and collectivization among the population. In the full, unweighted sample, FSWs were generally willing to self-identify as a sex worker—78.5 % strongly agreed or agreed that they were not ashamed to say they are a sex worker in meetings with other sex workers and 79.1 % strongly agreed or agreed that they were not ashamed to tell a social worker or health worker in their community that they are a sex worker. The women reported strong self-efficacy for their ability to use condoms with clients but slightly less confidence in their ability to utilize reproductive health services. Over 60 % felt very or completely confident that they could use condoms even when a client gets angry (61.4 %) or when a client offers more for sex without a condom (64.1 %), while only 55.4 % felt very or completely confident in their ability to use condoms with clients if they themselves had been using alcohol or drugs. Only 45.7 % felt very or completely confident in their ability to go to a government health clinic for reproductive health services if they thought the health workers would treat them poorly, and only 41.9 % felt very or completely confident in their ability to do so if the health workers knew they were a sex worker.

The women reported a sense of autonomy in making personal decisions. They did not need permission from someone else for going to a movie (66.5 %), purchasing new clothes (72.0 %) or participating in NGO activities (82.5 %). Likewise, they did not need permission to use contraception (68.0 %) or go to the doctor (69.4 %). Only 18.7 % reported usually or always turning away clients when tired, however. Just over a quarter reported feeling down, depressed or hopeless more than half the days over the past 2 weeks (26.9 %).

On the whole, FSWs reported a strong sense of collectivization amongst themselves. Over half the women attended a public event over the past 6 months in which they could be identified as a sex worker (55.7 %). In a 12-item measure of social cohesion on a 4-point scale from strongly disagree to strongly agree, women generally felt they could share with and rely on fellow sex workers for support (*m* = 2.96). Nearly half of the women (46.8 %) also reported that most or all FSWs would work together to address a problem that affected some or all the group. Over half felt very or completely confident that FSWs would work together to keep each other safe from harm (58.5 %), increase condom use with clients (81.8 %), speak up for their rights (70.6 %), and improve their lives (65.6 %). Furthermore, nearly half reported negotiating with or standing up to a police officer (42.6 %) or a madam or broker (42.6 %) during the past 6 months in order to help a fellow sex worker. Fewer women reported actually coming together, however, to demand access to entitlements such as voter ID cards (13.2 %), ration cards (14.2 %), or health insurance (17.3 %).

### Consistent Condom Use

Consistency of condom use with clients (occasional and regular) was operationalized as a three-category ordinal variable from low to high. Sixty-one percent of the propensity weighted sample reported high consistency of condom use, meaning they used condoms at every sex act with both regular and occasional clients. Condom use consistency had an intraclass correlation (ICC) of 0.18, indicating about 18 % of the variance occurred at the cluster level. This degree of cluster-level variation is consistent with other community and school intervention studies, which commonly have ICCs in the 0.05–0.20 range [34–36].

For our model, the two cluster-level covariates (duration of the Avahan program and density of FSWs in each cluster) were constrained to be independent of the cluster-level predictor and mediator components. Mediator components were freed to correlate at both the between- and within-cluster level. Tested models were thus nearly saturated, producing a close fit to the data as would be expected (Tucker-Lewis index = 1.00, RMSEA = 0.002, SRMR_within_ = 0.00, SRMR_between_ = 0.00).

**Fig. 3:**
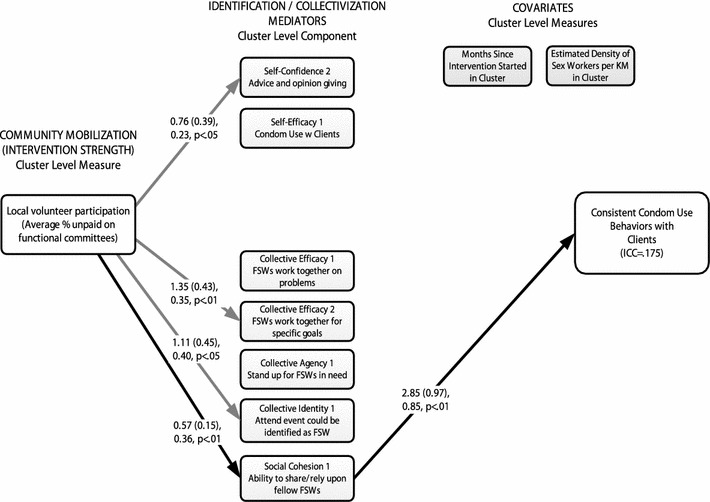
Cluster-level path model results for condom use with clients. Only *significant paths* shown; mediators were allowed to correlate freely in the model; *curved paths* are not shown for clarity. Regression estimates are reported as: unstandardized regression coefficient (standard error), standardized regression coefficient, *p*-level. *Solid paths* indicate significant effects (*p* < 0.05). *Darker solid paths* represent significant mediated (indirect) effect

Strength of community mobilization was positively associated with psychosocial mediators of identification and collectivization at the cluster level, including: increased self-confidence for advice/opinion giving (b = 0.76, SE = 0.39, β = 0.23, *p* < 0.05), increased collective identity (b = 1.11, SE = 0.45, β = 0.40, *p* < 0.05), increased collective efficacy for working toward specific goals (b = 1.35, SE = 0.43, β = 0.35, *p* < 0.01), and increased social cohesion (b = 0.57, SE = 0.15, β = 0.36, *p* < 0.01). Social cohesion was positively related to increased rates of consistent condom use with clients at the cluster level (b = 2.85, SE = 0.97, β = 0.85, *p* < 0.01). There was a significant indirect effect of community mobilization on consistent condom use mediated through social cohesion (b = 1.63, SE = 0.75, β = 0.31, *p* < 0.05); yet the relatively high standard error for the path from social cohesion to consistent condom use would suggest that this link be interpreted cautiously (Fig. 3). None of the other hypothesized mediators were significant, and no direct effect of community mobilization strength on the degree of consistent condom use was found.

### Perceived Discrimination

Perceived discrimination was reverse coded, and renamed perceived fairness. It was operationalized as a 4-item composite variable (Cronbach’s α = 0.80) on a 4-point scale representing perceptions of being treated not at all fairly to completely fairly in public places. Perceived fairness had an ICC of 0.37, indicating that about 37 % of the variance in perceived fairness occurred at the cluster level. The model again had a close fit to the observed data given the unconstrained correlations among mediator components (Tucker-Lewis index = 1.00, RMSEA = 0.001, SRMR_within_ = 0.00, SRMR_between_ = 0.00).

**Fig. 4:**
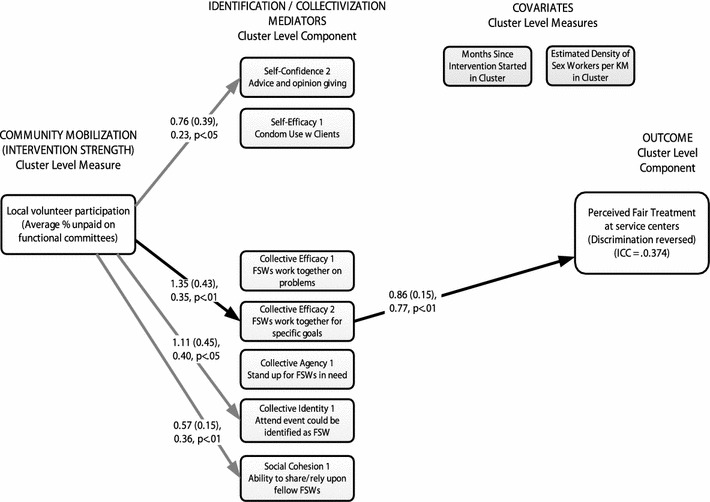
Cluster-level path model results for perceived fairness. Only *significant paths shown*; mediators were allowed to correlate freely in the model; *curved paths* are not shown for clarity. Regression estimates are reported as: unstandardized regression coefficient (standard error), standardized regression coefficient, *p*-level. *Solid paths* indicate significant effects (*p* < 0.05). *Darker solid paths* represent significant mediated (indirect) effects

As in the model for condom use, strength of community mobilization was positively associated with self-confidence for advice giving (b = 0.76, SE = 0.39, β = 0.23, *p* < 0.05), collective identity (b = 1.11, SE = 0.45, β = 0.40, *p* < 0.05), and social cohesion (b = 0.57, SE = 0.15, β = 0.36, *p* < 0.01) (Fig. 4).

Strength of community mobilization also had a positive effect on collective efficacy (b = 1.35, SE = 0.43, β = 0.35, *p* < 0.01) which was itself related to levels of perceived fairness (b = 0.86, SE = 0.15, β = 0.77, *p* < 0.01). This yielded a significant mediated effect of community mobilization on perceived fairness through collective efficacy (b = 1.16, SE = 0.43, β = 0.27, *p* < 0.01). None of the other psychosocial variables mediated the relationship between community mobilization and perceived fairness.

## Discussion

Community mobilization is a component of many HIV prevention programs, and yet little research has been done to evaluate whether or not it significantly contributes to improved outcomes, and, if so, what the mechanisms of effect are. In this study, we demonstrate the added value of community mobilization on key outcomes in an HIV prevention intervention for FSWs. All clusters received the same core intervention activities, yet the level of community mobilization varied widely across clusters, and clusters with stronger community mobilization had more positive HIV prevention outcomes. Not only do we show improved outcomes in geographic clusters with a higher level of community mobilization, but we begin to untangle the mechanism of effect, i.e. how the intervention works through key psychosocial factors of identification and collectivization that are influenced by mobilization.

We used a number of psychosocial scales to measure the processes of identification and collectivization within the FSW population. Many of our scales showed good reliability in this population, including those for self-efficacy, individual agency, social cohesion, mental health, collective efficacy, collective agency and collective action.

To our knowledge, this is the first time the previously validated 2-item depression screener [27] has been used with this population, and it showed strong reliability in our sample (Cronbach’s α = 0.88). Interestingly, the FSWs in our study reported relatively low levels of depression (*m* = 2.98 reverse coded, range 1.0–4.0), compared to a recent study from Goa, India in which 19 % of FSWs surveyed reported attempting suicide within the past 3 months [37]. Although our results cannot confirm this, we believe that community mobilization may provide some protective effects on mental health among FSWs, specifically through increasing social cohesion. The social cohesion scale we adapted [26] showed slightly lower reliability in our sample (Cronbach’s α = 0.69) than in the original Lippman study (Cronbach’s α = 0.81); however the FSWs from Andhra Pradesh, India in our study reported higher levels of social cohesion (*m* = 2.96) than did FSWs in urban Brazil (*m* = 1.55).^2^ This higher level of reported social cohesion may reflect the success of the Avahan community mobilization intervention in building a sense of trust, belongingness, and reciprocity among FSWs.

In both our models of consistent condom use and of perceived fair treatment in public places, the mediation effects that were observed both occurred through collectivization type variables: greater social cohesion was associated with increased consistency of condom use, and increased collective efficacy was associated with higher levels of reporting being treated fairly in public places. Our models suggest that these variables may be an important part of the mechanism through which community mobilization produces added value to intervention outcomes. Further disentangling the mechanisms by which community mobilization works to enhance outcomes is vital to improving HIV prevention programming.

Our study is the first to develop an independent measure of community mobilization strength in order to test the relationship between community mobilization and HIV prevention outcomes, including individual perceptions of discrimination in various public settings and consistent condom use with clients. We created a measure of community mobilization—ratio of volunteer FSW participation on program committees—that we believe indicates a deeper level of engagement with and investment in the intervention than simply numbers of participants in program activities. Despite confining this first phase of our evaluation to a specific region within one state, served by a single implementing partner responsible for the same set of core intervention activities within the clusters, the level of community mobilization varied widely.

Using an independent measure of community mobilization strength and adjusting for potential bias due to non-random distribution of participants into clusters with varying levels of treatment, model results suggest that Avahan’s community mobilization intervention does have positive indirect effects on FSW rates of consistent condom use and perceived discrimination above and beyond what would be expected due to the core interventions. The effects of community mobilization appear stronger for the enabling environment outcome of perceived discrimination, a social environmental characteristic, than for consistent condom use, an individual-level behavior. Since 37 % of the variance in perceived discrimination exists at the cluster-level versus only seventeen percent for consistent condom use, there was less variance in consistent condom use available at the cluster level to be explained by our model.

Given the intensity of Avahan’s targeted intervention activities, including community outreach and condom distribution, it is reasonable to expect that those interventions (outreach and condom distribution) would have a more profound effect on condom use than would high levels of engaged participation in the program (i.e. community mobilization). Still, community mobilization aims to help FSWs become more able to make decisions, influence their environment, and shape their lives in health enhancing ways, so we would expect some effect on condom use. The mediating influence of social cohesion on condom use appears to represent that effect, suggesting that a greater sense of belonging, trust and reciprocity among sex workers supports the use of condoms.

As predicted in our model, mediation effects occurred through the collectivization variables of social cohesion and collective efficacy for working together towards common goals, suggesting an important role for these constructs in producing positive program effects. The importance of collectivization is consistent with other studies of Avahan that show collective identity, efficacy, and agency associated with consistent condom use [21, 23, 38].

Still, our model of how community mobilization works to produce better outcomes is only partially validated. We predicted that community mobilization would catalyze a process of identification as a sex worker and increase self-efficacy and agency. Although the intervention was positively associated with these identification variables, they did not mediate the relationship between strength of community mobilization and consistent condom use or perceived discrimination. This suggests that change in the degree of identification may be necessary to change collectivization but not sufficient alone for influencing positive outcomes at the cluster level. Interestingly, while strength of the community mobilization intervention was positively related to collective identity, collective efficacy, and social cohesion, there was no significant relationship between strength of the intervention and measures of taking action on behalf of one another—collective agency and collective action. Perhaps these effects will emerge over time.

Our findings suggest that community mobilization may contribute to the success of Avahan by increasing the degree of social cohesion among FSWs which in turn increases rates of consistent condom use with clients. Community mobilization is also positively associated with levels of collective efficacy among FSWs, which in turn is associated with increased perceptions of fair treatment in public places such as banks, hospitals, and post offices.

## Limitations

Drawing from our program theory, we conducted a cross-sectional evaluation of model constructs for which we were able to construct valid measures. Not all of our measures fully captured the constructs of interest, however. For example, our measure of strength of community mobilization assessed level of volunteer participation of the FSWs in program committees as an indication of FSWs participation, engagement, commitment and ownership of the program—key characteristics of a “mobilized community”. Other measures of mobilization, such as the length and type of participation in the program (e.g. as an office holder), frequency of participation, and success of the various program committees in effecting change in the community, are not captured by our measure. Finally, though we weighted the sample to account for the time-location sampling procedures and the inverse propensity score weighting adjusted for bias associated with the 18 variables we identified, we cannot adjust for potential bias from unmeasured sources.

## Future Directions

Much has been written about Avahan recently. A population-level assessment estimated that Avahan averted over 100,000 HIV infections in India from 2003 to 2008 [39], and a more recent study suggests that infections averted are linked to large increases in condom use since implementation of Avahan [40]. Other studies have found associations between self-reported program exposure to Avahan and condom use within a single district in Andhra Pradesh, India [21, 22, 41]. We took a new approach to evaluating the program by focusing on the added value of community mobilization, as measured independently at the cluster-level.

Because community mobilization is both time and resource intensive, we believe it is important that programs explicitly evaluate the value added, even though this is difficult. Many believe that community mobilization improves program outcomes and long-term sustainability of those outcomes, but an evidence-base to support these assumptions must be established. As one part of building this evidence-base, our model of the relationship between community mobilization strength and HIV prevention outcomes should be validated across other SLPs in Avahan and across the different target populations of the program.

To better understand the mechanisms by which community mobilization enhances health outcomes (not just HIV prevention) and which intervention components are most essential for, and efficient at, spurring community mobilization, future interventions should plan carefully for the evaluation of the community mobilization process and outcomes, including the collection of baseline and non-intervention area data. Furthermore, more robust measures of community mobilization need to be developed. Measures of empowerment that include changes in relationship dynamics between community members and power holders, and more nuanced measures of participation and confidence in ability to influence power holders, would all enhance our understanding of how community mobilization works. We look forward to future investigations in these areas.

## Footnotes

1 HIV/AIDS Alliance was the state lead implementing partner.

2 Calculated from reported mean sum divided by reported number of items.
